# Numerical Design and Optimization of High Performance Langasite and Hetero-Acoustic Layer-Based Surface Acoustic Wave Device

**DOI:** 10.3390/mi16020166

**Published:** 2025-01-30

**Authors:** Minglong Deng, Jinkai Chen, Jikai Zhang, Weilun Xie, Hao Jin, Weipeng Xuan, Shurong Dong, Jikui Luo

**Affiliations:** 1 Ministry of Education Key Laboratory of RF Circuits and Systems, Hangzhou Dianzi University, Hangzhou 310018, China; 2 College of Information Science and Electronic Engineering, Zhejiang University, Hangzhou 310027, China; 3 International Joint Innovation Center, Zhejiang University, Jiaxing 314400, China

**Keywords:** surface acoustic wave, hetero-acoustic layer, harsh environment, numerical analysis, langasite

## Abstract

La_3_Ga_5_SiO_14_ (langasite, LGS)-based surface acoustic wave (SAW) devices are widely used for industrial health monitoring in harsh high-temperature environments. However, a conventional LGS-based SAW structure has a low quality factor (Q) due to its spurious resonant peaks. A hetero-acoustic layer (HAL)-based structure can effectively enhance the Q factor and the figure of merit (FOM) of SAWs due to its better energy confinement of SAWs. In this work, a HAL-based structure is proposed to achieve a high FOM and high-temperature resistance at the same time. Based on the finite element method (FEM) and coupling-of-model (COM) combined simulation, a systematic numerical investigation was conducted to find the optimal materials and structural parameters considering the viability of an actual fabricating process. After optimizing the layer number, an intermediate-layer material choice and structural parameters, Pt/(0°, 138.5°, 27°) LGS/YX-LGS/SiC HAL structure were chosen. The proposed structure achieves a Q factor and FOM improvement of more than 5 and 2.6 times higher than those of conventional SAW structures, which is important for the development of high temperature SAW sensors. These findings pave a viable method for improving the Q factor and FOM of LGS-based SAW and can provide material and device structural design guidance for fabrication and high-temperature applications in the future.

## 1. Introduction

With the development of electronic technology, surface acoustic wave (SAW) devices play an increasingly important role in the field of information and communication because of their characteristics of wireless and passive properties, especially in harsh environments such as aerospace and aviation, where the high-temperature and high-pressure environment makes conventional sensors difficult to handle. Among the existing high-temperature surface acoustic wave sensors, SAW devices based on ZnO and GaN typically operate at temperatures below 500 °C [1,2] due to their low curie temperatures. The commonly used piezoelectric materials are AlN and La_3_Ga_5_SiO_14_ (langasite, LGS) when sensing temperature is higher than 500 °C. Among them, the SAW sensor with AlN as the piezoelectric material can work under 900 °C for a long time, and has attracted the attention of many researchers because of its high acoustic wave velocity and high electromechanical coupling coefficient (*K*^2^) [3,4]. However, AlN begins to oxidize after more than 700 °C in the air environment, resulting in the wireless transmission ability of the corresponding device being weaker than that of LGS [5,6]. Therefore, researchers have been devoted to the study of LGS. The melting point of LGS is 1470 °C, and the phase transition will not occur before reaching the melting point. The electromechanical coupling coefficient of LGS can also reach 2–3 times of that of quartz, and the phase velocity is low at about 2700 m/s [7,8], which facilitates the effective miniaturization of devices. These characteristics make LGS an ideal material for the fabrication of high temperature SAW devices. Based on LGS, when the temperature, gas concentration, and stress experienced by the SAW sensor change, its frequency will drift. We can calculate the value of a certain environmental variable based on frequency change, thus, we can design corresponding pressure [9], gas [10], and temperature sensors. Xue et al. fabricated a pressure sensor capable of having a sensitivity of 137 Hz/kPa at 1000 °C [11], Zhou et al. made the designed temperature sensor work stably at 1100 °C by covering the LGS with a layer of AlN [12], and Zhang et al. developed a LGS–SAW sensor with a multilayer composite electrode structure, and the sensor can achieve wireless temperature measurements from room temperature to 1200 °C with an accuracy of 1.59% [13]. Nevertheless, the Q factor of LGS-based SAW devices is significantly lower than that fabricated on quartz substrate due to its spurious resonant peaks [14,15].

Therefore, this work is aiming at increasing the Q factor of LGS-based SAW devices by structure optimization. The hetero-acoustic layer (HAL)-based structure can effectively reduce energy leakage to the substrate and then enhance the Q factor and the figure of merit (FOM) defined by the Q factor and *K*^2^ of SAW devices, while simultaneously reducing the temperature coefficient of frequency (TCF) [16]. The incredible high performance (IHP) SAW resonator designed by Tsutomu et al. based on a LiTaO_3_/SiO_2_/AIN/Si structure can achieve 3 times of the Q of the conventional structure [17]. Hsu et al. utilized a LiNbO_3_/SiO_2_/Si structure to achieve a noticeable enhancement in both the *K*^2^ and Q factor compared with conventional SAW devices, with improvements of at least 1.15 times and 2 times for the *K*^2^ and Q factor, respectively [18]. Huo et al. designed an IHP–SAW device based on the LiTaO_3_/Polyimide/Si structure, achieving a 1.4 times and 4.4 times increase in the Q factor of the resonance and anti-resonance frequency, respectively [19]. These findings demonstrate that the use of a HAL structure can significantly enhance the performance of SAW devices.

In this work, a high Q, high-temperature resistant LGS-based HAL–SAW device was proposed, and a systematic numerical investigation was conducted to find the optimal structural parameters considering the viability of actual fabricating process. The device performances under different layer configurations, material choices, and layer thicknesses have been simulated and compared. Finally, a Pt/(0°, 138.5°, 27°) LGS/YX-LGS/SiC-based HAL structure shows the best performance, and the simulated Q factor and FOM are found to be 5 and 2.6 times greater than those of conventional SAW devices, respectively. This design improves the performance of LGS-based SAW sensors significantly, which provides new insights for the development of high-performance LGS-based SAW devices.

## 2. Structural Design of LGS Based HAL SAW Device

### 2.1. Simulation Methods

#### 2.1.1. COM Model

**Figure 1:**
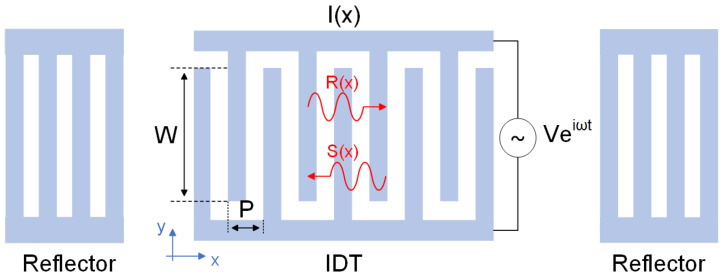
Schematic diagram of the COM model configuration for one-port resonator.

The coupling-of-model (COM) model is the mainstream simulation approach for investigating the performance of SAW devices, which is accurate and efficient [20]. A classic structure of a one-port SAW resonator is shown in Figure 1, where *W* and *P* is the aperture and the pitch of grating, respectively.

In the COM model, the AC driving voltage loaded on the bus bar excites two counter-propagating acoustic wave modes *R*(*x*) and *S*(*x*), These modes couple to each other through the interdigital reflection effect of the metal grating, then those two waves generate a current on the interdigital transducer (IDT) due to the piezoelectric effect, which is expressed by *I*(*x*). After considering the effects such as propagation loss and electrode reflection, the governing equation of COM can be obtained as follows [21,22]:

(1) $$\left\{\begin{array}{c} \frac{dR\left(x\right)}{dx}=j\kappa S\left(x\right)\exp(2j\Delta x)+j\alpha V\exp(j\Delta x)\\ \frac{dS\left(x\right)}{dx}=-j\kappa^{*}R\left(x\right)\exp(-2j\Delta x)-j\alpha^{*}V\exp(-j\Delta x)\\ \frac{dI\left(x\right)}{dx}=-2j\alpha^{*}R\left(x\right)\exp(-j\Delta x)-2j\alpha S\left(x\right)\exp(j\Delta x)+j\omega CV \end{array}\right.$$

where *κ* indicates the reflection coefficient, *α* represents transduction coefficient(superscript * denotes the complex conjugate), *C* is the static capacitance, and Δ denotes the detuning parameter, which is defined by:

(2) $$\Delta =\frac{\omega }{v}-k_{0}-j\gamma$$

where *ω* is calculate by ω=2πf, *f* is frequency, *v* represents velocity of the SAW, the *k_0_* is the synchronous wave number and the *γ* is the propagation attenuation. By obtaining these parameters, the propagation characteristics of the SAW can be obtained. The normalized independent parameters *κ*, *α*, *C*, *v*, and *γ* will be determined in Section 2.1.2.

#### 2.1.2. The Extraction of COM Model Parameters

**Figure 2:**
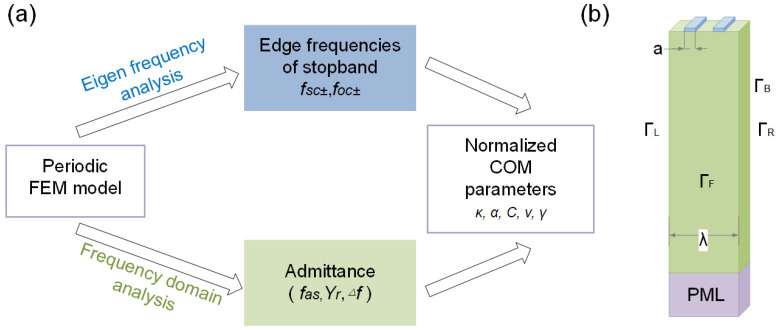
(**a**) The process of the COM model parameter extraction based on the (**b**) unit cell model of the resonator.

As the COM model is a phenomenological model, the extracted parameters should be as accurate as possible to ensure reliable simulation results of SAW devices [23]. Here, the finite element method (FEM) software COMSOL Multiphysics 6.0 was used to simulate and calculate the extracted parameters for the COM model precisely [24,25]. The specific process is shown in Figure 2a, Since the IDT structure used in SAW devices has some periodicity, in order to save calculation time, only one transduction period structure was taken for simulation. As shown in Figure 2b, periodic boundary conditions were set on the left (Γ_L_), right (Γ_R_), front (Γ_F_), and back (Γ_B_) of the model, and a perfectly match layer (PML) was added to reduce the reflection from the bottom. In this paper, the metallization ratio (MR=2a/λ) was set to 0.5, so the width of electrode *a* is *λ*/4.

When the electrode is set to a short-circuit condition, that is, one electrode is set to ground and the other is applying 1 V voltage, *f_sc-_* and *f_sc+_* can be obtained through eigen frequency analysis, which represents the lower edge and upper edge of stopband at short-circuited grating, respectively. In the same way, the lower edge *f_oc-_* and upper edge *f_oc+_* at open-circuited grating can be obtained. Subsequently, the *v* and *κ* can be determined as follows:

(3) $$f_{0}=\frac{\left(f_{sc+}+f_{sc-}\right)}{2}$$

(4) $$v=\lambda f_{0}$$

(5) $$\left|\kappa \right|=2\pi \frac{f_{sc+}-f_{sc-}}{f_{sc+}+f_{sc-}}$$

The admittance *Y* can be calculated based on frequency domain analysis of the proposed model, then, the *γ* and *C* at resonance frequency can be extracted as follows:

(6) $$\gamma =\pi \frac{\Delta f}{f_{0}}$$

(7) $$C=\frac{2Y_{r}Q\left(\frac{f_{ar}}{f_{sc-}}-1\right)}{2\pi f_{ar}\left[4Q^{2}{\left(\frac{f_{ar}}{f_{sc-}}-1\right)}^{2}+1\right]}$$

where Δ*f* represents the 3dB bandwidth of conductance *Y_r_*, *f_ar_* is the anti-resonance frequency, and the Q factor of the FEM model is defined by $Q=f_{sc-}/∆f$. Finally, the *α* can be obtained using the following:

(8) $$\left|\alpha \right|=\sqrt{\omega C\lambda \pi \left(\frac{f_{oc+}+f_{oc-}}{f_{sc+}+f_{sc-}}-1\right)}$$

By bringing the calculated COM model parameters into the P-matrix [26], the device performance can be calculated accurately. For example, *K*^2^ can be calculated from the edge frequencies *f_sc±_* and *f_oc±_* of the stopband:

(9) $$K^{2}=\pi \frac{f_{oc-}+f_{oc+}-f_{sc-}-f_{sc+}}{f_{sc-}+f_{sc+}}$$

The overall device performance, characterized by the quality factor Q, can be calculated using the following equation:

(10) $$Q=\frac{f_{r}}{2}\left|\frac{d\phi }{df}\right|$$

where *f_r_* is the resonance frequency and *Φ* is the impedance phase, with the results obtained from the COM model. Then, the FOM can be calculated as follows, which characterizes the overall performance of the device, and we aim to maximize this value while ensuring that the Q factor and *K*^2^ are at normal levels [27]:

(11) $$FOM=K^{2}\times Q$$

### 2.2. Optimizing the Layer Number for LGS Based HAL SAW Device

Through the HAL structure, a better energy confinement can be achieved. For example, as the surface acoustic wave propagates in HAL–SAW devices, energy is reflected from the high acoustic impedance layer to the low acoustic impedance layer, thereby reducing energy leakage into the substrate and enhancing device performance [28]. In this work, based on the HAL structure with low-fast acoustic velocity variation, the energy confinement performances and frequency responses have been simulated using different layer-number configurations, and a comparison has been made to obtain the structure with an optimal layer number.

**Figure 3:**
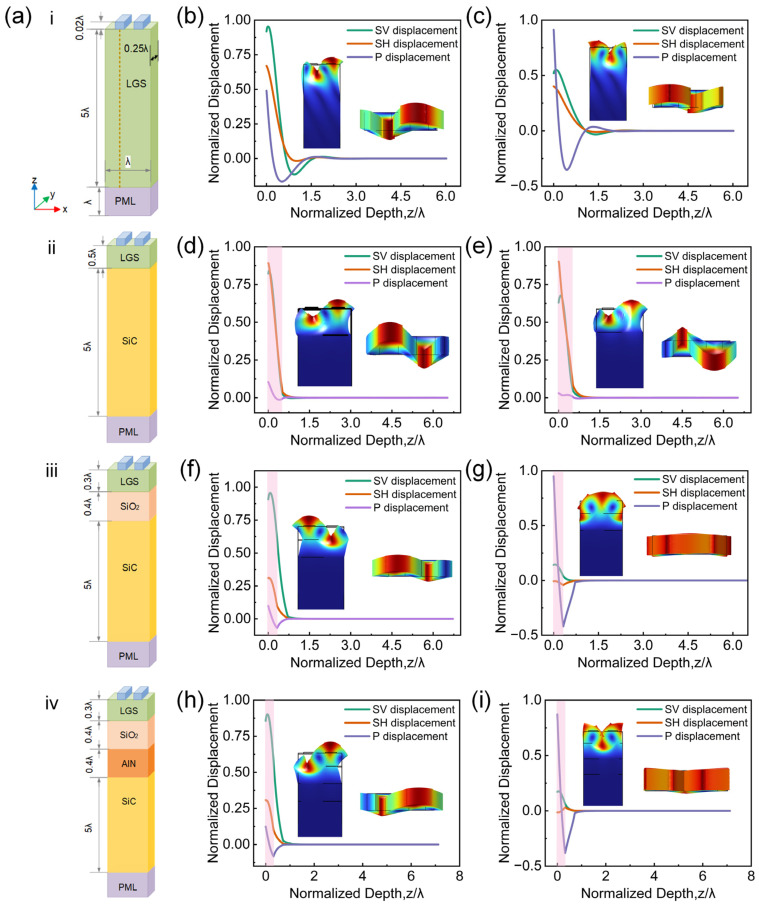
(**a**) FEM simulation model diagrams of (**i**) conventional structure, (**ii**) two-layer structure, (**iii**) three-layer structure, and (**iv**) four-layer structure. Displacement component and vibration mode diagrams for a conventional structure at (**b**) 435.13 MHz and (**c**) 437.69 MHz resonance frequency, for a two-layer HAL structure at (**d**) 458.96 MHz and (**e**) 517.67 MHz resonance frequency, for a three-layer HAL structure at (**f**) 426.39 MHz and (**g**) 422.04 MHz resonance frequency, and for a four-layer HAL structure at (**h**) 425.55 MHz and (**i**) 421.43 MHz resonance frequency.

Based on our previous experimental work [29], LGS with Euler angles of (0°, 138.5°, 27°) and Pt was selected as the piezoelectric material and electrode material, respectively, which allows stable operation at temperatures up to 1200 °C. First, a simulation was performed using a conventional structure as shown in Figure 3(ai). A cutting line was placed at the center of the electrode, the length direction is the z-axis direction, the displacement components at the cutting line and total displacement were observed at the resonance frequency, which are shown in Figure 3b,c for resonance frequency *f* at 435.13 MHz and 437.69 MHz, respectively. These two figures indicate that the vertical shear displacement (SV), horizontal shear displacement (SH), and longitudinal displacement (P) components are gradually decaying to zero within ~1.9 wavelengths.

Subsequently, simulation of a two-layer structure was performed. To ensure the device can operate at high temperatures, the substrate material must have both high acoustic velocity and excellent thermal stability. SiC fully meets these requirements, with a high shear wave velocity of 7250 m/s and a sublimation point of 2830 °C, as it does not have a defined melting point [30,31]. Additionally, the thermal expansion coefficients of SiC and LGS are similar, which is 5.1 ppm/°C and 5.6 ppm/°C [32,33], making them suited for high-temperature application. The simulation model is shown in Figure 3(aii). Figure 3d,e depict the vibration modes and displacement components at frequencies of 458.96 MHz and 517.67 MHz, respectively. The red shaded areas in the figures indicate the location of LGS thin films. In addition, it can be observed that the energy is highly confined in the LGS layer compared with the result from a conventional structure, as shown in Figure 3b,c, with the displacement components mainly confined within ~0.6 wavelengths. It should be noted that SAW-resonator structural design usually focuses on enhancing the transmission property based on one specific acoustic wave mode (e.g., Rayleigh or SH wave mode, etc.), and a clear distinction between different acoustic wave modes must be achieved beforehand. For example, generally speaking, the SH wave mode means that SH displacement is the main component, and the Rayleigh wave mode means that both SV and P displacements are the main component. However, the enhanced acoustic confinement induced by large differences of velocity between LGS and SiC, which is approximately 4500 m/s, results in the strong coupling between the Rayleigh mode wave and the SH mode wave, the vibration modes at the resonance frequencies in the two-layer structure model contain significant SV and SH components simultaneously, and their values are very close [34]. As a result, the vibration modes at these two different eigen frequencies are nearly identical, making it difficult to distinguish between the Rayleigh and SH wave modes. A similar situation is observed for other thickness configurations, which is unfavorable for device design and performance enhancement [35,36].

Then, the three-layer HAL structure was analyzed, where an additional dielectric layer was introduced between the SiC and LGS layers, as shown in Figure 3(aiii). Considering the high-temperature application, SiO_2_ is temporarily selected for this layer, as it has a melting point of 1750 °C and an acoustic velocity of approximately 3150 m/s, making it suitable for harsh environmental conditions [37]. The displacement components and vibration mode at the resonance frequency of 426.39 MHz and 422.04 MHz are shown in Figure 3f and Figure 3g, respectively. In the Figure 3f, the energy is concentrated around the LGS layer, with some transition into the SiO_2_ layer, but it remains confined within ~0.8 wavelengths. Moreover, the SV component is much larger than the SH component, and the Rayleigh wave mode can be clearly defined, which is advantageous for the subsequent simulation and design.

Furthermore, an additional high-temperature-resistant dielectric layer, AlN, was introduced between the SiO_2_ and SiC layers, forming a four-layer HAL structure as shown in Figure 3(aiv). As shown in Figure 3h,i at 425.55 MHz and 421.43 MHz, the vibration mode and displacement components are nearly identical to those in the three-layer structure, which confined within ~0.9 wavelengths, suggesting that the energy confinement in the four-layer structure is similar to that in the three-layer structure.

Based on the above simulation results, several conclusions can be drawn. First of all, a HAL structure leads to a better energy confinement. Secondly, although the energy is properly concentrated in the two-layer HAL structure, there is severe coupling between different acoustic modes, leading to a difficulty of obtaining the correct extracted parameters (e.g., *f_sc±_* and *f_oc±_*) used for the device structural optimization based on the COM model. Therefore, the three-layer and four-layer HAL structures are chosen for the subsequent optimization due to their significant energy confinement property and clear distinction between different acoustic modes.

### 2.3. The Selection of Intermediate-Layer Material

**Figure 4:**
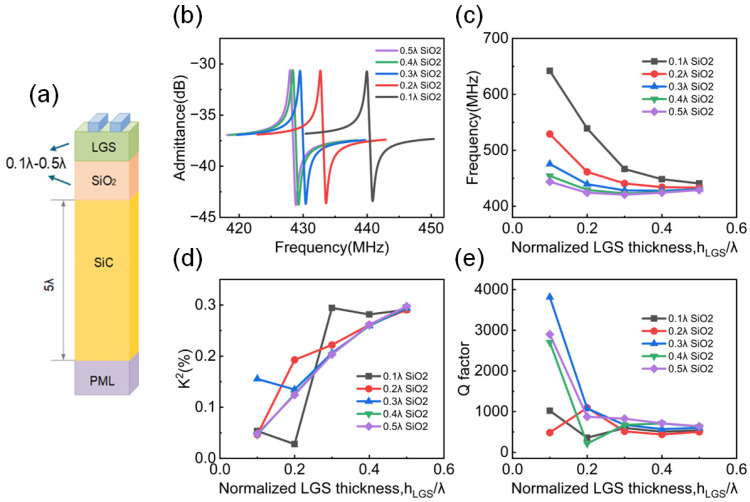
(**a**) Three-layer FEM simulation model diagram. (**b**) The admittance spectrum with 0.5*λ* LGS thickness and varying SiO_2_ thicknesses. The variation trend of (**c**) frequency, (**d**) *K*^2^, and (**e**) Q factor in the structure of Pt/LGS/SiO_2_/SiC with different LGS and SiO_2_ thicknesses.

Due to the anisotropy of the multilayer piezoelectric/dielectric material, surface acoustic waves propagating through the structure exhibit dispersion characteristics. Acoustic parameters such as wave velocity and *K*^2^ vary with frequency and the properties of the dielectric materials. The admittance spectrum is shown in Figure 4a with 0.5*λ* LGS thickness and varying SiO_2_ thicknesses. No discernible spurious peaks were observed between the resonance and anti-resonance frequencies. To achieve the optimal device performance, the impact of varying the thickness of each thin film under different material configurations on the device performance was investigated.

In the three-layer structure, when considering thermal stability as the primary criterion, there are several material options for the intermediate dielectric layer, such as AlN, SiO_2_, Al_2_O_3_, etc. All of these materials can withstand high temperatures. However, when AlN and Al_2_O_2_ are used as the intermediate-layer materials, they exhibit relatively high acoustic velocities compared to LGS, for instance, the shear wave velocity of Al_2_O_3_ can reach 6045 m/s [38]. Although the energy is highly concentrated in the LGS layer, the vibration modes of the resonance frequencies obtained from the simulation are similar to those of the two-layer structure in Figure 3d,e, both of the resonance frequencies exhibit significant SV and SH displacement components simultaneously, making it difficult to easily distinguish between the different acoustic modes.

In Section 2.2, the frequency analysis on the three-layer structure with SiO_2_ as the intermediate layer have already been performed. The results show that this structure offers high energy confinement and clearly distinguishable acoustic modes. A more detailed study of the performance for this structure will now be proceeded with. The electrode thickness is still set to 0.02*λ*. Other structure parameters are shown in Figure 4a. As shown in Figure 4c, the HAL structure has a certain effect on influencing the device frequency. Generally, with the increase in LGS thickness, the resonance frequency experiences a decreasing trend. Additionally, as the SiO_2_ thickness increases from 0.1*λ* to 0.5*λ*, the frequency also decreases and eventually stabilized with 0.5*λ* SiO_2_ thickness. Different with the frequency behavior, as shown in Figure 4d, when the thickness of LGS increases, the electromechanical coupling coefficient *K*^2^ also increases. In contrast, changes in the thickness of SiO_2_ have little effect on *K*^2^. It should be noted that fluctuations can be observed when the SiO_2_ thickness is relatively small, which is due to the enhancement of acoustic-wave-modes coupling when the piezoelectric layer is in close proximity to the high velocity layer SiC, and this fluctuation will be stable after the thickness is larger than 0.4*λ*.

According to Figure 4e, as the thickness of LGS increases, the Q factor decreases, and stabilizes when the thickness of LGS reaches 0.4*λ*. The thickness of the SiO_2_ film also has an effect on the Q factor, but it does not follow a monotonic trend, with the maximum Q factor occurring when the thickness of SiO_2_ is 0.3*λ*.

Based on the structural optimization with SiO_2_ as the intermediate layer, high-performance devices can be obtained. For example, when the LGS thickness is 0.3*λ* and the SiO_2_ thickness is 0.4*λ*, the Q factor can reach nearly three times that of conventional devices with the same electrode thickness. However, considering that the thermal expansion coefficient of LGS is approximately 5 ppm/°C, while the thermal expansion coefficient of SiO_2_ is highly dependent on its crystalline form [39,40,41], in most cases, the thermal expansion coefficient of SiO_2_ differs from that of LGS by more than 2 ppm/°C. The mismatch between the two materials may lead to significant thermal stress at high temperatures, potentially causing the LGS film to crack, ultimately resulting in device failure. To avoid such problems, the intermediate-layer material must have a thermal expansion coefficient similar to that of LGS. Based on the above simulation results, a conclusion can be induced that the intermediate-layer material must possess the following properties at the same time: (1) high thermal stability; (2) high acoustic velocity; (3) a thermal expansion coefficient similar to LGS. Materials that meet all these requirements are scarce. However, since LGS with different orientations exhibits different acoustic velocities [42], which may be the most suitable candidates for the intermediate-layer material. For example, the shear wave velocity of YX-cut LGS is approximately 2300 m/s, which is 400 m/s less than the (0°,138.5°,27°) LGS, while other parameters remain largely consistent. In terms of the fabrication process, some preliminary investigations have already been conducted by other researchers, proving the viability of making an LGS-based HAL–SAW device. For example, Pei et al. report that the single crystal-single crystal (dual-SC) bonding of two LGS wafers is achieved through thermal activation under a low compression of 50 kPa, and the bonding strength has reached 23 Mpa [43]. Wulfmeier et al. successfully deposited LGS thin films with a defined crystal structure on LGS and Si substrates, achieving a thickness of 3 µm [44].

**Figure 5:**
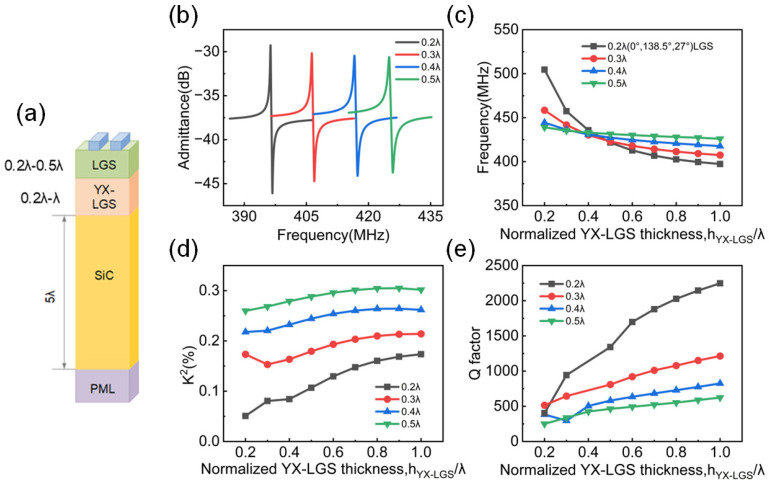
(**a**) Three-layer FEM simulation model diagram. (**b**) The admittance spectrum with *λ* YX-LGS thickness and varying (0°,138.5°,27°) LGS thicknesses. The variation trend of (**c**) frequency, (**d**) *K*^2^, and (**e**) Q factor in the structure of Pt/(0°,138.5°,27°)LGS/YX-LGS/SiC.

Therefore, YX-cut LGS was selected as the intermediate-layer material for structural optimization as shown in Figure 5a. Subsequently, as shown in Figure 5b, when the YX-LGS thickness is *λ*, the admittance spectrum has no spurious peaks, and it can be clearly seen that with the increase in the thickness of the first LGS layer, the amplitude of admittance is gradually decreasing and the distance between the resonance frequency and anti-resonance frequency is increasing, which represents the change in *K*^2^. The two layers of LGS with different orientations jointly affect the frequency. As shown in Figure 5c, when the thickness of (0°,138.5°,27°) LGS is less than 0.2*λ*, the frequency decreases with the increasing thickness of YX-cut LGS and then stabilizes after reaching a certain thickness. However, when the (0°,138.5°,27°) LGS thickness exceeds 0.2*λ*, the influence of the YX-cut LGS thickness on the frequency decrease becomes gradual, and when the (0°,138.5°,27°) LGS layer thickness reaches 0.5*λ*, the effect of changing the YX-cut LGS thickness on the frequency becomes negligible. Similar to that behavior, as shown in Figure 5d, as the thickness of the YX-cut LGS increases, *K*^2^ gradually increases. Moreover, a thicker (0°,138.5°,27°) LGS layer results in a larger *K*^2^. However, a thicker (0°,138.5°,27°) LGS layer results in a smaller *Q* factor, as shown in Figure 5e. As the thickness of the YX-cut LGS increases, the Q factor also increases. However, as the thickness of the first LGS layer increases, the influence of changes in the YX-cut LGS thickness on the Q factor diminishes, and the Q factor gradually stabilizes. Therefore, in Figure 5e, a trade-off must be considered when choosing a proper thickness of (0°,138.5°,27°) LGS layer.

**Figure 6:**
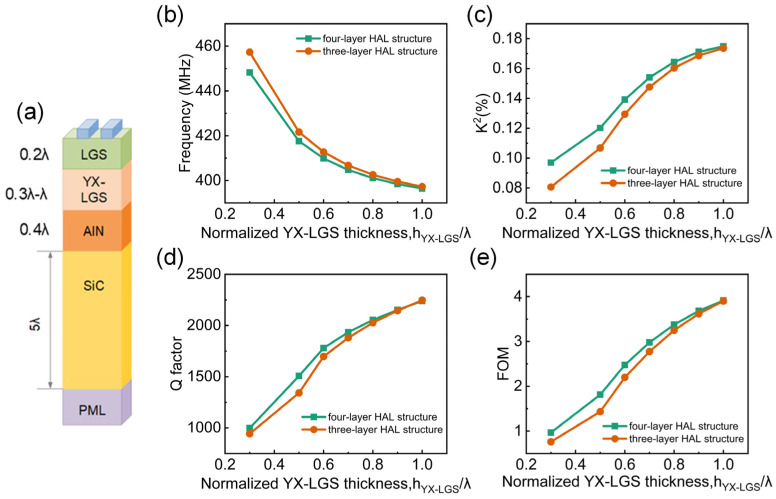
(**a**) FEM Simulation model diagram of the four-layer HAL structure. Comparison of (**b**) frequency, (**c**) *K*^2^, (**d**) Q factor, and (**e**) FOM between three-layer and four-layer HAL structures with varied thickness of YX-LGS.

In the three-layer HAL structure, significant tuning effects have already been observed. To further optimize the performance, an AlN layer was added between the YX-cut LGS and SiC layers in the three-layer HAL structure, resulting in a four-layer structure: Pt/(0°, 138.5°, 27°) LGS/YX-LGS/AlN/SiC. Based on the current simulation results of the three-layer structure, the overall device performance was compared using the FOM. It was found that the device achieves the maximum FOM when the thickness of the (0°,138.5°,27°) LGS layer is 0.2*λ*. Therefore, in the four-layer structure, the Pt electrode thickness was fixed at 0.02*λ* and the other structural parameters are shown in Figure 6a. The performance of the device was studied as the thickness of the YX-cut LGS layer varied from 0.3*λ* to *λ*, and the results were compared with the three-layer structure. As the thickness of the YX-cut LGS layer increases, as shown in Figure 6b, the frequency of the three-layer structure is slightly higher than that of the four-layer structure, and both frequencies decrease as the thickness of the YX-cut LGS increases. As shown in Figure 6c,d, the *K*^2^ and the Q factor of both structures increase with the thickness of the YX-cut LGS. The maximum *K*^2^ value for the four-layer HAL structure is 0.173%, which is nearly identical to the maximum *K*^2^ value of 0.174% for the three-layer HAL structure. Meanwhile, the maximum Q factor obtained for the four-layer structure is 2230, which is almost the same as the maximum value of 2247 for the three-layer structure. Finally, comparing the FOM of both structures, as shown in Figure 6e, the results indicate that their trends are generally consistent, with the four-layer structure being only slightly better than the three-layer structure.

In summary, the conclusion can be drawn that the tuning effect of the four-layer HAL structure on SAW devices with LGS as the piezoelectric material is nearly identical to that of the three-layer HAL structure. Considering the increased processing complexity and cost associated with adding more layers, the three-layer HAL structure is sufficient to achieve the desired performance enhancement for the device, and three-layer HAL structure is used for the subsequent structural optimization.

### 2.4. Electrode Thickness Optimization and Performance Comparison Between Conventional and HAL Structure SAW Devices

**Figure 7:**
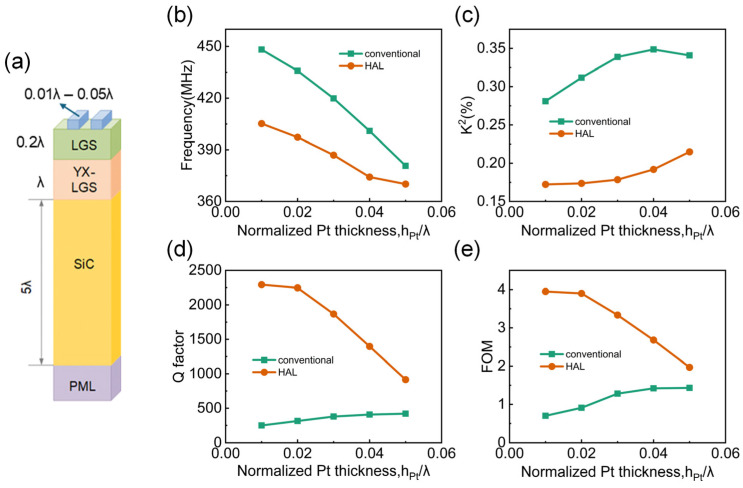
(**a**) FEM simulation model diagram of the three-layer HAL structure with varying electrode thickness. Comparison of (**b**) frequency, (**c**) *K*^2^, (**d**) Q factor and (**e**) FOM from the conventional and HAL structure SAW devices with varied Pt electrode thicknesses.

Based on the current simulation results, the device exhibits optimal performance when the (0°, 138.5°, 27°) LGS thickness is small and the YX-cut LGS thickness is large, although the frequency is minimal in this situation. To further enhance the device performance, this study also investigates the impact of varying the Pt electrode thickness on the device’s performance, and the FEM 3D structure is shown in Figure 7a. The corresponding changes in frequency, *K*^2^, and Q factor were calculated and compared with the conventional structure. As shown in Figure 7b, due to the significant mass loading effect induced by the Pt electrodes [45], the device frequency decreases sharply as the Pt electrode thickness increases, with a maximum reduction of 68 MHz, and the device frequency in this HAL structure is slightly lower than that of the conventional structure, but, its decrease trend is slower than that of conventional structures. Unlike the frequency variation, as shown in Figure 7c, *K*^2^ increases with the electrode thickness in both structures. Although the *K*^2^ value in the conventional structure is nearly twice that in the HAL structure, this does not affect the higher overall performance of the HAL structure. As shown in Figure 7d, the Q factor of the device in the conventional structure remains below 500 over all the electrode thickness variations, whereas in the HAL structure, although the Q factor decreases more rapidly with increasing electrode thickness, even when the electrode thickness reaches 0.05*λ*, the Q factor in the HAL structure is still twice that of the conventional structure. Subsequently, the resulting FOM for the HAL structure at smaller electrode thickness is also significantly higher than that of the conventional structure, as shown in Figure 7e.

**Figure 8:**
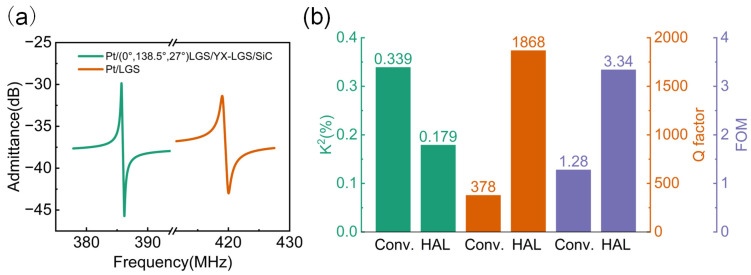
The comparison of (**a**) admittance spectrum and (**b**) performance parameters between optimized HAL and conventional structure SAW devices.

At last, considering both the device’s performance and its service life, the electrode thickness was set to 0.03*λ* to ensure a proper thermal stability and avoid excessively high resistance caused by an extremely thin electrode. The comparison of admittance at the resonance frequencies of two structures is shown in Figure 8a. Consistent with the results in Figure 7, the resonance frequency of the HAL structure is lower than that of the conventional structure. Additionally, the distance between the resonance and anti-resonance frequency is also smaller in the HAL structure compared to the conventional one. However, the amplitude of the HAL structure is significantly greater than that of the conventional structure. The detailed performance parameters for this structure, along with a comparison to the conventional structure, are shown in Figure 8b. Although the *K*^2^ value is lower than that of the conventional structure, the Q factor in the HAL structure is nearly five times larger. Ultimately, the FOM is 2.6 times greater than that of the conventional structure, showing a significant improvement. It is worth noting that although materials with similar coefficients of thermal expansion have been selected, the maximum temperature the device can withstand is still influenced by the material quality and the manufacturing process. Consequently, significant thermal stress may arise at high temperatures, potentially leading to device failure.

## 3. Conclusions

This paper presents numerical investigation of a high-temperature LGS and HAL structure-based SAW device, employing FEM- and COM model-based methods. The study investigates the effects of different layer numbers, intermediate-layer materials, and thicknesses of films on the device performance. It is determined that a three-layer HAL structure can achieve high performance, with the intermediate layer needing to meet the criteria of high-temperature resistance, acoustic velocity, and the thermal expansion coefficient similar to that of LGS. Finally, an optimal structure of Pt/(0°, 138.5°, 27°) LGS/YX-LGS/SiC is obtained, and the performance changes with varying Pt electrode thicknesses are simulated and compared with the conventional LGS-based SAW device structure, which shows a 5-fold increase in Q factor and a 2.6-fold increase in FOM, providing a viable solution to the problem of significant Q factor degradation in LGS-based SAW sensors at high temperature. This work offers important insights for material and device structural design guidance for fabrication and high-temperature applications in the future.

## Data Availability

Data will be made available on request.
